# A survey of topical treatment adherence and self-compassion in people living with psoriasis

**DOI:** 10.1093/skinhd/vzag100

**Published:** 2026-06-23

**Authors:** Elaine N Clarke, Rachael J Thorneloe

**Affiliations:** Centre for Behavioural Science and Applied Psychology, Sheffield Hallam University, Sheffield, UK; Centre for Behavioural Science and Applied Psychology, Sheffield Hallam University, Sheffield, UK

## Abstract

This study investigated topical treatment adherence and self-compassion in people living with psoriasis. The results of a cross-sectional survey of 130 participants showed that people who were more self-compassionate tended to be more adherent with their prescribed topical psoriasis treatment.

Dear Editor, Nonadherence to prescribed topical treatments for psoriasis is a common issue.^1^ In other medical populations, being more self-compassionate is linked with higher medication adherence.^2^ Self-compassion is a multifaceted construct centred around being supportive of oneself in times of distress.^3^ The link between treatment adherence and self-compassion has not yet been investigated among people living with psoriasis; therefore, we conducted an online cross-sectional survey to address this research gap.

Participants were recruited online following review by a university ethics committee. Participants were recruited via adverts on social media and/or the websites of the Psoriasis Association, the Psoriasis and Psoriatic Arthritis Alliance, the British Association of Dermatologists and the British Skin Foundation. Adverts were also circulated on Facebook and Twitter (now X) from the researchers’ institutional accounts and the Psoriasis Association publicized information about the study via their members’ newsletter, which included a QR code. Participants were eligible to take part in the study if they were aged ≥18 years and self-reported being prescribed one or more topical therapies for psoriasis with an active ingredient (e.g. topical steroids, tar preparations and vitamin D analogues). The survey contained demographic and clinical questions, a measure of treatment adherence [the Medication Adherence Report Scale-5 (MARS-5),^4^ adapted for a topical treatment context by referring to ‘using topical treatment’ rather than ‘taking medication’] and two measures of self-compassion [the Self-Compassion Scale (SCS)^5^ and the self-compassion scale of the Compassionate Engagement and Action Scales (CEAS-SC)^6^]. Further details of the survey questions can be found in Appendix S1 (see Supporting Information). In our sample, several of the continuous variables were non-normally distributed, so medians and interquartile ranges (IQRs) are reported to better describe these data.

Most (*n* = 110; 84.6%) of the 130 participants were of White British ethnicity, 66.9% (*n* = 87) were women and 60.0% (*n* = 78) were educated to degree level or above. Most (*n* = 87; 66.9%) participants were employed, 18.5% (*n* = 24) were retired, 7.7% (*n* = 10) were disabled or unable to work, 2.3% (*n* = 3) were homemakers or carers, 2.3% (*n* = 3) were students, 1.5% (*n* = 2) were currently unemployed and one participant (0.8%) chose not to report their employment status. The types of psoriasis and prescribed treatments are shown in Figure 1. Participants could fall into more than one category for these two variables. Participants most commonly had plaque psoriasis, and the most commonly prescribed type of active topical therapy was topical steroids. Most (*n* = 92/130; 70.8%) participants had been prescribed topical treatments only and 29.2% (*n* = 38) had also been prescribed ultraviolet light therapy, systemic treatments, or biologic or biosimilar treatments. The median age of onset of psoriasis was 20.0 years (IQR 12.5–32.0), the median duration of psoriasis was 20.6 years (IQR 10.0–38.0) and the median duration of the current topical treatment was 6.0 years (IQR 2.8–14.5).

**Figure 1:**
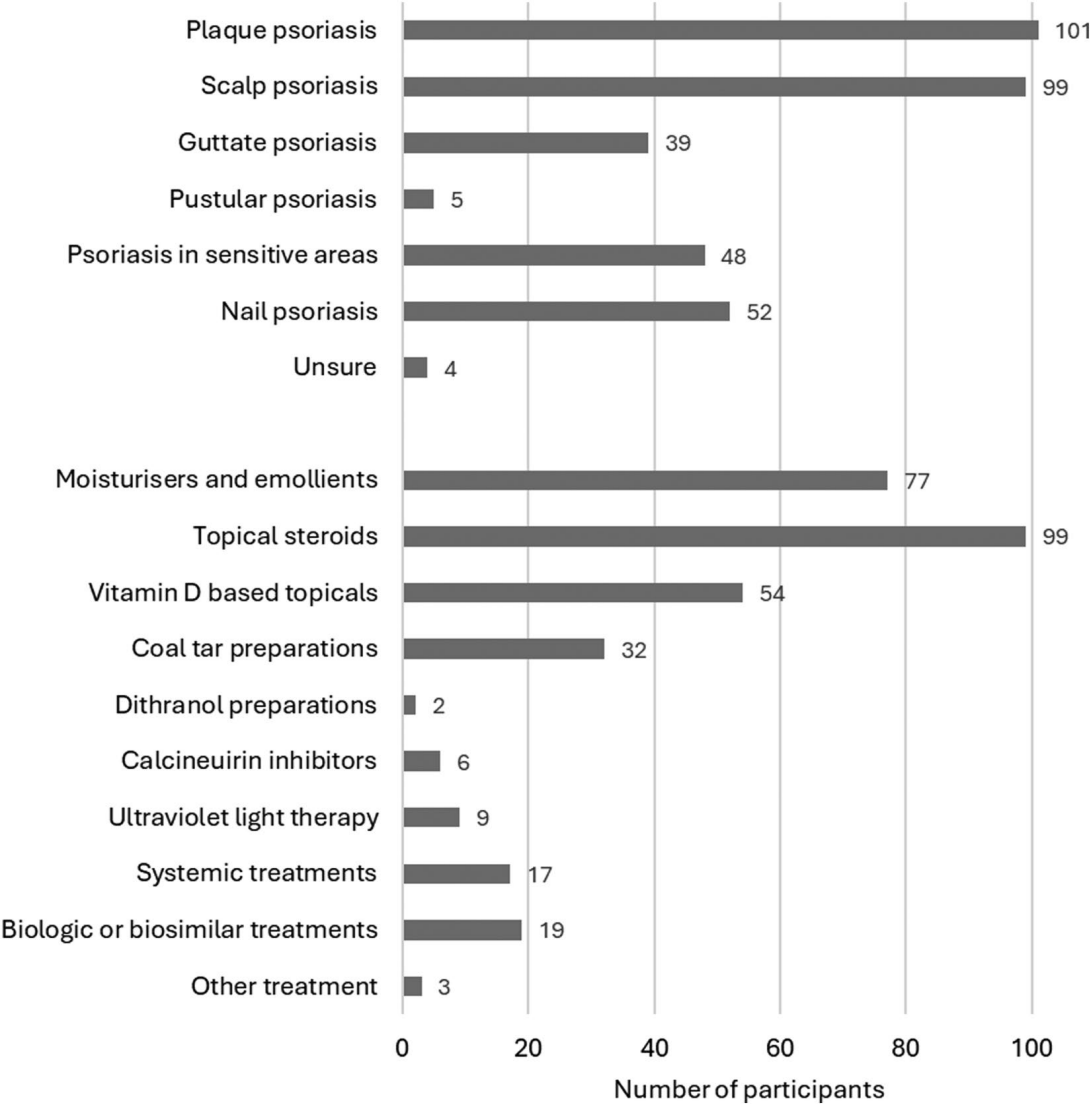
Type of psoriasis and prescribed treatments.

The mean (SD) treatment adherence score (scale range 5–25) was 14.92 (4.34). The median total SCS score was 2.83 (IQR 2.29–3.47), with median subscale scores of 2.80 (IQR 2.20–3.40) for self-kindness, 3.00 (IQR 2.50–3.75) for common humanity, 3.25 (IQR 2.50–3.75) for mindfulness, 3.40 (IQR 2.60–4.20) for self-judgement, 3.25 (IQR 2.50–4.00) for isolation and 3.25 (IQR 2.50–3.75) for overidentification (all scale ranges 1–5). Scores for self-judgement, isolation and overidentification were reverse coded for calculating the SCS total score. The median CEAS-SC total score (scale range 10–100) was 60.0 (IQR 48.0–70.0), with median subscale scores of 35.0 (IQR 30.0–42.0) for engagement (scale range 6–60) and 25.0 (IQR 18.0–30.0) for action (scale range 4–40).

Pearson correlation analyses were used to investigate whether treatment adherence varied with continuous variables. Independent *t*-tests and one-way independent Anovas were used to investigate whether treatment adherence differed with cat­egorical variables. Bias corrected and accelerated (BCa) interval bootstrapping using 10 000 bootstrap samples was used as a robust test to overcome problems of non-normal data. Analyses were carried out in SPSS 26 (IBM, Armonk, NY, USA). BCa bootstrap 95% confidence intervals (CIs) are reported.

Treatment adherence did not significantly vary with age, gender, ethnic background, education level, employment status, psoriasis duration or current topical therapy duration (all *P* > 0.05). However, treatment adherence was significantly positively associated with age at onset of psoriasis [*r*(123) = 0.29, 95% CI 0.10–0.45; *P* = 0.001], indicating that adherence to prescribed topical treatment regimens tended to increase with the age at which participants developed psoriasis.

Self-compassion, as measured by the SCS, was significantly positively associated with treatment adherence [*r*(121) = 0.19, 95% CI 0.01–0.38; *P* = 0.02], while compassion for self, as measured by the CEAS-SC, was not associated with treatment adherence [*r*(116) = 0.06, 95% CI −0.14 to 0.25; *P* = 0.27]. Five of the SCS subscales were found to be significantly associated with treatment adherence: self-kindness was positively associated with treatment adherence [*r*(121) = 0.15, 95% CI −0.04 to 0.33; *P* = 0.05], as was mindfulness [*r*(121) = 0.17, 95% CI −0.03 to 0.36; *P* = 0.03], while self-judgement was negatively associated with treatment adherence [*r*(121) = −0.15, 95% CI −0.30 to 0.04; *P* = 0.05], as were isolation [*r*(121) = −0.19, 95% CI −0.36 to −0.001; *P* = 0.02] and overidentification [*r*(121) = −0.21, 95% CI −0.40 to −0.01; *P* = 0.01]. However, the 95% CIs for the associations between treatment adherence and self-kindness, mindfulness and self-judgement included zero, reducing confidence in the significance of these effects, despite the significant *P*-values. Neither the remaining SCS subscale (common humanity) nor the two CEAS-SC subscales (engagement and action) were significantly associated with treatment adherence (all *P* > 0.05). The reason for the discrepancy in the results between the two measures of self-compassion is unclear. Although measuring similar constructs, it is possible that the different wording and format of the items contributed to this effect. The SCS presents each item without reference to any preamble, for example ‘When something upsets me I try to keep my emotions in balance’, and it varies the phrases used to describe instances of suffering. In contrast, the CEAS-SC prefaces all items with the phrase ‘When I’m distressed or upset by things…’ and only uses the term ‘distress’ in its items, for example, ‘I reflect on and make sense of my feelings of distress’. In a general population sample, it may have been easier for participants to relate everyday opportunities for self-compassion to the wording in the SCS rather than the CEAS-SC. However, further research would be needed to investigate this possibility.

Our results indicate that people with greater self-compassion (as measured by the SCS) tend to be more adherent to their prescribed topical psoriasis treatment. This is consistent with the proposal that self-compassion facilitates health-promoting behaviours.^3,7^ The small positive correlation found between self-compassion and treatment adherence aligns with the findings of previous research investigating this relationship in other medical populations (average *r* = 0.22).^2^ Many different factors can affect topical treatment adherence,^8^ and in this complex context, modifiable factors such as self-compassion that are only modestly associated with treatment adherence may still provide meaningful benefits.

Our study has limitations. Firstly, we used a correlational design, which means that strong inferences about causality cannot be made, although there is evidence that increasing self-compassion leads to increases in health behaviours and physical health.^9^ Secondly, we used a sample that was self-selected from nonclinical settings, which probably led to a sample of people with above-average interests in psoriasis self-management, so these findings require replication in clinical settings. Nevertheless, our study is the first demonstration that self-compassion is linked with better treatment adherence in people living with psoriasis and suggests that self-compassion may be a useful therapeutic target in interventions to support adherence.

## Supplementary Material

vzag100_Supplementary_Data

## Data Availability

The data underlying this article will be shared on reasonable request to the corresponding author.
