# Supplementary material for: A survey of topical treatment adherence and self-compassion in people living with psoriasis
Source: Skin Health Dis. 2026 Jun 23;6(4):638–40. doi: 10.1093/skinhd/vzag100 (PMC13425025; doi:10.1093/skinhd/vzag100)
Supplement: vzag100_Supplementary_Data [file vzag100_supplementary_data.docx]

**Supporting Information: Survey Questions**

Please answer the following questions.

Q1 What is your age? [open text box]

Q2 What is your gender identity?

- Male
- Female
- Non-binary
- Prefer not to say

Q3 Which of these best describes your ethnic identity?

- White - British
- White - Irish
- White - Any other White background
- Mixed - White & Black African
- Mixed - White & Black Caribbean
- Mixed - White & Asian
- Mixed - Any other Mixed background
- Black or Black British - Caribbean
- Black or Black British - African
- Black or Black British - Any other Black background
- Asian or Asian British - Indian
- Asian or Asian British - Pakistani
- Asian or Asian British - Bangladeshi
- Asian or Asian British - Chinese
- Asian or Asian British - Any other Asian background
- Any other ethnic background
- Prefer not to say

Q4 What is your highest level of education?

- No formal qualifications
- O-level/GCSE/BTEC equivalent
- GCE/A-level/BTEC equivalent
- Degree
- Postgraduate Degree
- Other (please state) [open text box]
- Prefer not to say

Q5 What is your employment status?

- Employed full-time
- Employed part-time
- Unemployed
- Student
- Full-time homemaker or carer
- Retired
- Disabled or unable to work
- Prefer not to say

Q6 How long have you had psoriasis (in years or months as appropriate, enter 0 as needed)?

- Years [open text box]
- Months [open text box]

Q7 What type of psoriasis do you have? Select all that apply.

- Plaque psoriasis
- Scalp psoriasis
- Guttate psoriasis
- Pustular psoriasis
- Psoriasis in sensitive areas
- Nail psoriasis
- Unsure

Q8 At what age did you develop psoriasis? [open text box]

Q9 What treatments are you **currently** prescribed for your psoriasis? Select all that apply.

- Moisturisers and emollients
- Vitamin D based topicals
- Topical steroids
- Coal tar preparations
- Dithranol Preparations
- Calcineurin Inhibitors
- Ultraviolet Light Therapy
- Systemic treatments (tablets, e.g., Methotrexate, Ciclosporin, Acitretin)
- Biologic or biosimilar treatments (injections, e.g., Adalimumab, Etanercept, Infliximab)
- Other (please state) [open text box]

Topical treatments are the ones you apply directly to your skin (creams, ointments etc). The following questions are about the topical treatments you have been prescribed that have an 'active' ingredient (e.g., vitamin D based topicals, topical steroids, coal tar preparations, dithranol preparations, calcineurin inhibitors).

Q10 How long have you been prescribed your current topical treatments with an active ingredient (in years or months as appropriate)? If you are prescribed more than one, please tell us about the one you have been prescribed the longest.

- Years [open text box]
- Months [open text box]

Q11 < Medication Adherence Report Scale-5 (MARS-5) ©Professor Rob Horne.

See Chan AHY, Horne R, Hankins M, Chisari C. The Medication Adherence Report Scale: A measurement tool for eliciting patients’ reports of nonadherence. Brit J Clinical Pharma. 2020 Jul;86(7):1281–8. <https://doi.org10.1111/bcp.14193>

Adapted with permission for the context of topical psoriasis treatments: “Thinking about your active topical treatment(s) for psoriasis (not including moisturisers or emollients) and how your doctor has asked you to use it, for each statement, please select the answer which best applies to you.” Statements were amended to use the verb ‘to use’ rather than ‘to take’.

For full conditions of use and permission to use the scale, contact Prof. Horne at [r.horne@ucl.ac.uk](mailto:r.horne@ucl.ac.uk).>

Q12 The next set of questions asks about how you typically act towards yourself in difficult times.

< Self-Compassion Scale.

See Neff K. The Development and Validation of a Scale to Measure Self-Compassion. Self and Identity. 2003a;2(3):223–50. <https://doi.org/10.1080/15298860309027>

Scale available at <https://self-compassion.org/self-compassion-scales-for-researchers/> >

Q13 The next set of questions is about the degree to which you can be compassionate with yourself.

< The Compassionate Engagement and Action Scale – Compassion for Self scale.

See Gilbert P, Catarino F, Duarte C, Matos M, Kolts R, Stubbs J, et al. The development of compassionate engagement and action scales for self and others. J of Compassionate Health Care. 2017 Dec;4(1):4. <https://doi.org/10.1186/s40639-017-0033-3>

Scale available at <https://www.compassionatemind.co.uk/resource/scales> >

That is the end of the survey questions.
